# Haplotype phasing after joint estimation of recombination and linkage disequilibrium in breeding populations

**DOI:** 10.1186/2049-1891-4-30

**Published:** 2013-08-06

**Authors:** Luis Gomez-Raya, Amanda M Hulse, David Thain, Wendy M Rauw

**Affiliations:** 1 Departamento de Mejora Genética Animal, Instituto Nacional de Investigación y Tecnología Agraria y Alimentaria (INIA), Ctra. de La Coruña km 7, 28040, Madrid, Spain; 2 Interdisciplinary Program in Genetics, Texas A&M University, College Station, TX 77843, USA; 3 Department of Animal Biotechnology, University of Nevada, 1664 North Virginia Street, Reno, NV 89557, USA

**Keywords:** Breeding, Haplotype phasing, Linkage disequilibrium, SNP

## Abstract

A novel method for haplotype phasing in families after joint estimation of recombination fraction and linkage disequilibrium is developed. Results from Monte Carlo computer simulations show that the newly developed E.M. algorithm is accurate if true recombination fraction is 0 even for single families of relatively small sizes. Estimates of recombination fraction and linkage disequilibrium were 0.00 (SD 0.00) and 0.19 (SD 0.03) for simulated recombination fraction and linkage disequilibrium of 0.00 and 0.20, respectively. A genome fragmentation phasing strategy was developed and used for phasing haplotypes in a sire and 36 progeny using the 50 k Illumina BeadChip by: a) estimation of the recombination fraction and LD in consecutive SNPs using family information, b) linkage analyses between fragments, c) phasing of haplotypes in parents and progeny and in following generations. Homozygous SNPs in progeny allowed determination of paternal fragment inheritance, and deduction of SNP sequence information of haplotypes from dams. The strategy also allowed detection of genotyping errors. A total of 613 recombination events were detected after linkage analysis was carried out between fragments. Hot and cold spots were identified at the individual (sire level). SNPs for which the sire and calf were heterozygotes became informative (over 90%) after the phasing of haplotypes. Average of regions of identity between half-sibs when comparing its maternal inherited haplotypes (with at least 20 SNP) in common was 0.11 with a maximum of 0.29 and a minimum of 0.05. A Monte-Carlo simulation of BTA1 with the same linkage disequilibrium structure and genetic linkage as the cattle family yielded a 99.98 and 99.94% of correct phases for informative SNPs in sire and calves, respectively.

## Background

Advances in molecular biology have allowed rapid and massive genotyping of Single Nucleotide Polymorphisms (SNP) in animal and plant species. SNP arrays have been implemented for genome-wide selection to enhance genetic improvement of farm animals [1]. In many instances, it has been done without consideration of the nature of the SNP information. That is, SNPs have been assumed to be unlinked in spite of providing low information (maximum PIC values of 0.375 at intermediate frequencies for two alleles). In reality, SNP polymorphisms are arranged in aligned sequences forming haplotypes where the order of the alleles for consecutive SNPs within each homologue chromosome contains relevant information. To make full use of SNP microarray technology, haplotype phasing must be estimated because genotyping only generates information for each single SNP. Haplotype phasing consists of arranging the order of allelic variants in a chromosomal segment within each of the two homologous chromosomes of diploid species. Phasing knowledge can be applied to trace SNP inheritance and to account for regions that are identical by descent in genomic evaluations aimed at the genetic improvement of agricultural species [2,3]. Haplotype phasing can be assessed in the laboratory or computationally [4]. Computational methods can make use of the population structure of linkage disequilibrium assuming no relationships among individuals or may also establish inheritance of haplotypes within pedigrees in order to reconstruct haplotypes. In the latter, haplotypes can be traced up to ancestors in the top of the pedigree [5-7]. PHASE [7], FASTPHASE [6], and BEAGLE [5] implement a Bayesian statistical method for reconstructing using coalescent and hidden Markov models. FASTPHASE [6] cannot provide estimates of recombination rates but PHASE [7] does. BEAGLE [5] can infer haplotypes from unrelated individuals, parent-offspring pairs, and parent-offspring trios. Rohde and Fuerst [8] developed methods for haplotype inference based on maximum likelihood, which could be used with nuclear families. Their method was based on searching long genomic segments for which an allele was shared for two individuals and has been also used for phasing haplotypes. A linkage program like GENEHUNTER can extract multiple relative’s information [9], although it is done by assuming linkage equilibrium between polymorphisms, which may lead to incorrect phasing. If the genotype information is from sibs and genotyping information on one of the parents is missing then linkage disequilibrium (from gametes from the other parent) may not be separated from genetic linkage (within the parent with genotype information). Another group of methods makes use of relative’s information [10,11] together with laws of Mendelian inheritance to infer haplotypes. Haplotypes inferred from data sets from families are accurate across extended genomic distances if family sizes are large. Williams *et al.*[12] proposed a new haplotype inference method for nuclear families based on maximum likelihood and minimum recombination using linear programming (implemented in software Hapi). More recently, Lai *et al.*[13] proposed an algorithm for reconstructing haplotypes in a pedigree by assuming zero recombinants. Their method can be applied to general pedigrees but it is not designed to make use of nuclear families with one single parent.

All of the above methods for haplotype inference cannot be readily applied to the situation most commonly found in farm animals. This is, genotyping information is available from just one of the parents which in turn have large progeny groups (e.g., dairy bulls with up to one million progeny). Therefore, none of the above methods can estimate recombination and linkage disequilibrium simultaneously because they assume that there is linkage equilibrium. As stated by Browning and Bronwing [4]: “When sites are in LD, linkage programs that assume linkage equilibrium may falsely infer IBD in situations in which is not present”.

In this study, we develop a computational method to estimate haplotype phases in breeding populations using closely linked biallelic markers densely distributed in the entire genome. The strategy is based on making use of large families of breeding populations in order to estimate recombination fraction and linkage disequilibrium (LD) simultaneously in order to phase parents. Then, progeny are phased using Mendelian laws of inheritance together with genotyping information from parents and progeny as it has been done elsewhere e.g., [9-11]. A new EM algorithm is developed to simultaneously estimate recombination fraction and linkage disequilibrium in half-sib families (required for accurate phasing). The method to phase haplotypes is tested in a cattle family with 36 calves. Montecarlo-computer simulations were carried out for joint estimation of recombination and linkage equilibrium in a family resembling recombination and linkage disequilibrium estimated from real data in BTA1.

## Methods

### Genome fragmentation phasing strategy

Assume SNP microarrays with a dense coverage of the animal genome are used for large-scale genotyping of animals. The situation considered is dairy cattle in which bull sires and bull dams are mated (usually by artificial insemination) and have usually one progeny from each dam (half-sibs). Among the progeny of those elite bulls are young bulls that are typed for microarrays in order to carry out pre-selection based on genomic information. Some of the bull dams are also progeny of elite bulls and this process repeats itself in successive generations. Therefore, all male selection candidates are typed for microarrays. A method named genome fragmentation phasing strategy (GFPS) is proposed to phase haplotypes when information is available in families as it is common in breeding populations. GFPS steps assumptions are: 1) families are large with progeny sharing at least one parent; 2) both linkage disequilibrium and recombination events are modeled; and 3) use is made of SNP arrays with high coverage of the genome and with known SNP location. The steps for GFPS are:

A. ***Estimation of the recombination fraction in consecutive SNPs using family information in the first generation***

There are two possibilities depending on the available genotype information from one or from two parents. If both parents are available then standard linkage analyses can be performed. Non-informative progeny can be ignored and the resulting recombination fraction estimates will be unbiased. If only one parent is known then joint estimation of estimation of recombination fraction and linkage disequilibrium (LD) is needed for each pair of consecutive closely linked SNPs. An EM algorithm is proposed to estimate recombination and LD (Appendix 1). Monte-Carlo computer simulation was carried out to validate joint estimation of LD. If recombination fraction between two consecutive SNP, T/t and M/m, is zero then:

A1 *Establish linkage phase in the parent or parents by eq.A3:*

$$\begin{array}{l} \mathrm{Pr}\;\mathrm{of}\;\text{Phase}\;\left(\mathrm{TM}/\mathrm{tm}\right)\\ \;=\frac{L_{\mathrm{TM}/\mathrm{tm}}\left(\delta ,f_{T},f_{M},c|\mathrm{data}\right)}{L_{\mathrm{TM}/\mathrm{tm}}\left(\delta ,f_{T},f_{M},c|\text{data}\right)+L_{\mathrm{Tm}/\mathrm{tM}}\left(\delta ,f_{T},f_{M},c|\text{data}\right)} \end{array}$$

where *L*_*TM*/*tm*_(*δ*, *f*_*T*_, *f*_*M*_, *c*|data) and *L*_*Tm*/*tM*_(*δ*, *f*_*T*_, *f*_*M*_, *c*|data) are the likelihoods of phase (*TM/tm*) and phase (*Tm/tM*) under a model estimating linkage disequilibrium (*δ*), allele frequencies for one of the alleles at markers *T/t* and *M/m* (*f*_*T*_, and *f*_*M*_), and the recombination fraction (*c*). See Appendix 1 for a full description of the maximum likelihood method. Repeat this process for each pair of consecutive SNPs in which the last SNP in a pair is the first SNP of the following until an estimate of recombination fraction is greater than 0. If recombination fraction is greater than 0 then it means that either a recombination event has taken place or it is a genotyping error. This is a signal for the termination of one fragment and the starting of the next one. Genotyping errors can be of two kinds: failure of genotyping itself or incorrect alignment of SNPs in the array. At this point, all consecutive SNPs fully linked and aligned two by two are considered a fragment, or haplotype block.

A1 *Reconstruct phases of parent(s) for each fragment*

**Figure 1:**
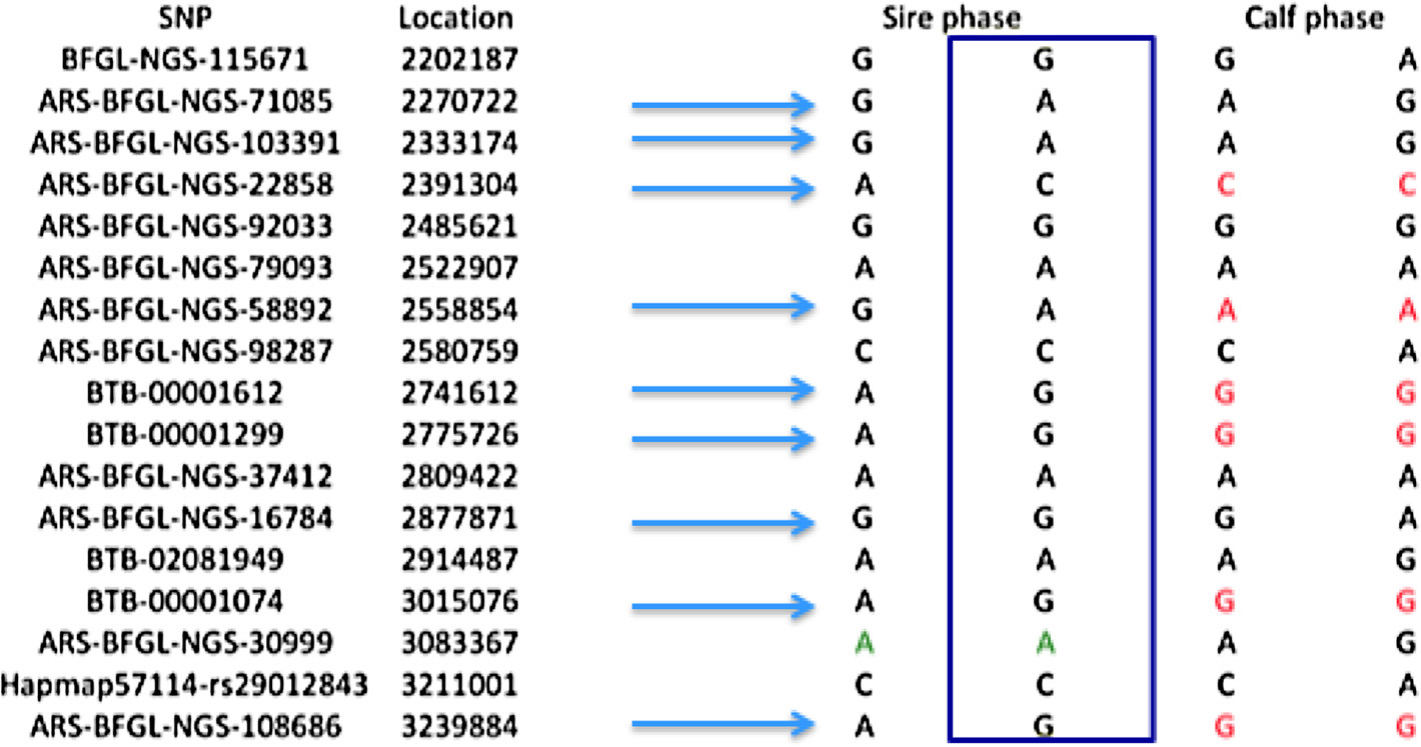
Scheme of the genome fragmentation phasing strategy using SNP information from a sire and one progeny of cattle.

The phases for each pair of SNPs in the sire are reconstructed by aligning the most likely phases for each pair of SNPs until fragment phasing information is completed. Therefore, a fragment is a piece of a chromosome in which no recombination events are detected between any consecutive markers. If the sire is homozygote then the same allele would appear in the two homologous chromosomes. Figure 1 illustrates reconstruction of parent haploypes for a sire. The blue arrow indicates heterozygote SNPs that are concatenated according the linkage phases having the higher probability.

A3 *Reconstruct phases in progeny with paternal and maternal haplotypes*

The sire has two fragments and the progeny has one paternal fragment and another maternal fragment that it is deducted from the genotype information. Progeny phases can be reconstructed by the following rules. If a progeny is homozygous for one of the alleles for which the parent is heterozygous then the full haplotype of that fragment is inherited by that progeny (square in the Figure 1). The contribution from the mother completes the genotype information for each SNP within the fragment. Homozygous calf’s genotypes from an heterozygous sire are represented in red in Figure 1. If the progeny is heterozygous at one SNP but the parent is homozygous then that paternal allele in the progeny is the sire allele while the maternal allele is the other allele (green in Figure 1).

B. ***Linkage analyses between fragments***

Fragments with no recombination events detected between SNPs can be used in linkage analyses considering long haplotypes as highly informative “super-alleles” (haplotypes of fragments whose inheritance can be traced to their paternal and maternal contributions), which allows detection of recombination events. Also changes in the haplotype configuration inherited in the calf when compared to sire’s phased chromosomes may reveal a recombination event in that calf.

C. ***Phasing of haplotypes in following generations***

Once the phase is established in the parents and progeny, phases of individuals in the following generation is straightforward. Phases of progeny that become a parent and contributes to the next generation are used to reconstruct phases in the progeny of the progeny. Linkage analyses are carried out in the next generation using family information in order to establish haplotype phases in each of the subsequent generations.

### Monte-Carlo computer simulation of a half-sib family for joint estimation of LD and genetic linkage

A Monte-Carlo computer simulation was carried out in order to validate methods to estimate recombination fraction and LD jointly in half-sib families. The sire was assumed to be a double heterozygote. A random generator from the uniform distribution was used to assign progeny with the genotypes *TTMM*, *TTMm*, *TTmm*, *TtMM*, *TtMm*, *Ttmm*, *ttMM*, *ttMm*, and *ttmm* according to their probability (frequency): ϕTTMM=121-cfTM, ϕTTMm=121-cfTm+12cfTM, ϕTTMm=12cfTm, ϕTTMm=121-cftM+12cfTM, ϕTTMm=121-cftm+fTM+12cftM+fTm, ϕTTMm=121-cfTm+12cftm, ϕTTMm=12cftM, ϕTTMm=121-cftM+12cftm, and ϕTTMm=121-cftm. If the drawing of the uniform distribution was between 0 and *ϕ*_*TTMM*_, then the offspring had genotype *TTMM*. If the drawing of the uniform distribution was between *ϕ*_*TTMM*_ and *ϕ*_*TTMM*__+_*ϕ*_*TTMm*_ then the offspring genotype was *TTMm*. Assigning other genotypes to offspring was done following the same rule.

### Multi-family estimation of linkage disequilibrium

A total of six families with sizes 94, 77, 106, 81, 79, and 100 half-sib progeny were simulated resembling the sire Norwegian cattle population (after pooling selected and culled bulls in Table 1 of [14]). The allele frequencies were intermediate, recombination fraction was 0, 0.25, or 0.50, and linkage disequilibrium ranged from 0 to 0.25. The sires were simulated as if they were coming from a population with the same linkage disequilibrium and allele frequencies as used to generate the half-sib progeny. In order to do so, the two haplotypes of each sire were generated following the same principles as above, with probabilities according to the simulated frequencies: *f*_*TM*_ = *δ* + *f*_*T*_*f*_*M*_, *f*_*Tm*_ = - *δ* + *f*_*T*_*f*_*m*_, *f*_*tM*_ = - *δ* + *f*_*t*_*f*_*M*_, and *f*_*tm*_ = *δ* + *f*_*t*_*f*_*m*_, in which allele frequencies *f*_*M*_, *f*_*m*_, *f*_*T*_, *f*_*t*_, and *δ* were input parameters. Thus, the sire could be a double homozygote, homo-heterozygote or double heterozygote after assigning the two haplotypes. The half-sib progeny was generated as described in the previous section. Join estimation of linkage disequilibrium and recombination fraction was carried out using the developed E.M. algorithm for multiple families (Appendix 1). Each experiment was replicated 10,000 times.

**Table 1:** **Average estimates of linkage disequilibrium in a double heterozygote sire with varying half-sib family size and varying recombination fraction (*c*) and linkage disequilibrium (*δ*)**

		**Family size**					
**Simulated *c***	**Simulated *δ***	**36**	**100**	**300**	**500**	**1,000**	**2,000**
**0.00**	-0.10	-0.0905 (0.0401)	-0.0998 (0.0228)	-0.1001 (0.0131)	-0.1001 (0.0101)	-0.1000 (0.0072)	-0.1000 (0.0051)
	0.00	-0.0044 (0.0412)	-0.0014 (0.0250)	-0.0007 (0.0143)	-0.0003 (0.0110)	-0.0000 (0.078)	-0.0001 (0.0056)
	0.05	0.0434 (0.0409)	0.0479 (0.0245)	0.0492 (0.0140)	0.0496 (0.0109)	0.0499 (0.0077)	0.0499 (0.0055)
	0.10	0.0914 (0.0392)	0.0973 (0.0232	0.0990 (0.0133)	0.0995 (0.0101)	0.0998 (0.0072)	0.0998 (0.0051)
	0.20	0.1873 (0.0304)	0.1957 (0.0163)	0.1985 (0.0089)	0.1992 (0.0068)	0.1996 (0.0048)	0.1998 (0.0034)
**0.10**	-0.10	-0.0958 (0.0540)	-0.0997 (0.0277)	-0.1003 (0.0153)	-0.1002 (0.0118)	-0.0999 (0.0084)	-0.1000 (0.0059)
	0.00	0.0076 (0.0069)	0.0042 (0.0422)	0.0002 (0.0192)	0.0001 (0.0134)	0.0001 (0.0099)	0.0000 (0.0000)
	0.05	0.0544 (0.0664)	0.0581 (0.0493)	0.0531 (0.0281)	0.0513 (0.0189)	0.0505 (0.0118)	0.0502 (0.0081)
	0.10	0.0925 (0.0576)	0.1081 (0.0493)	0.1074 (0.0346)	0.1059 (0.0281)	0.1032 (0.0197)	0.1013 (0.0125)
	0.20	0.1534 (0.0364)	0.1721 (0.294)	0.1856 (0.0262)	0.1882 (0.0224)	0.1891 (0.0178)	0.1909 (0.0144)
**0.20**	-0.10	-0.0889 (0.0705)	-0.0978 (0.0374)	-0.1001 (0.0184)	-0.1001 (0.0141)	-0.0999 0.0099	0.1000 (0.0071)
	0.00	0.0776 (0.0764)	0.0116 (0.0553)	0.0053 0.0340	0.0026 (0.0239)	0.0010 (0.0147)	0.0003 (0.0096)
	0.05	0.0485 (0.0723)	0.0572 (0.0535)	0.0580 (0.0384)	0.0569 (0.0322)	0.0549 (0.0246)	0.0525 (0.0167)
	0.10	0.0818 (0.0651)	0.0961 (0.0508)	0.1008 (0.0362)	0.1017 (0.0307)	0.1033 (0.0258)	0.1039 (0.0215)
	0.20	0.1237 (0.0461)	0.1474 (0.0425)	0.1526 (0.0316)	0.1536 (0.0274)	0.1538 (0.0222)	0.1539 (0.0184)

The simulated allele frequencies were ƒ_*T *_= 0.5 and ƒ_*M *_= 0.5. The values between parentheses are standard deviations among replicates.

### Programming source code for GFPS

Subroutines were written in Fortran 90 to compute join estimates of LD and recombination fraction using the E.M. algorithm as well as to perform all steps of the GFPS algorithm. All source code is available on request to the authors (gomez.luis@inia.es).

### Genome analyses of LD in a beef cattle half-sib family

A half-sib family consisting of 36 calves from commercial beef cattle at the Gund ranch in Nevada was used to illustrate and to compare alternative methods for estimation of linkage disequilibrium. The first step was to determine paternity of the calves at the ranch. A set of 25 microsatellites (BMS410, BMS499, BMS650, BMS1244, BMS1634, TGLA227, BMS601, BMS1789, BMS2005, ILSTS081, BMS1315, BMS1226, BMS2573, ILSTS058, TGLA126, CSSM66, SPS115, TGLA53, BM1824, BM2113, ETH3R, TGLA122, INRA023, ETH225, ETH10) was used to assign paternity that was carried out using Cervus software. The procedures are fully described in [15]. The Illumina bovine 50K BeadChip was used with bull #302 and his 36 calves in order to compare methods to estimate LD in half-sib families. Only SNPs with a call rate higher than 0.80 in at least 24 calves and with MAF of 0.10 or more were used. The data was also filtered for SNPs that were not consistent for inheritance from sire to progeny. If a SNP was not consistent for one progeny then the SNP information was discarded for the entire family. Only pairs of SNPs within the same chromosome and within a distance of 50Mb or less were used for estimating linkage disequilibrium and recombination fraction.

The GFPS algorithm was used to reconstruct haplotypes of fragments in the sire and its 36 calves. Recombination events between fragments were detected. In addition, regions of identity (ROI) were used as a measure of molecular relatedness. ROIs are a generalization of runs of homozygosity (ROH) and are the proportion (in respect to the total of the genome) of long haplotypes shared by two individuals. ROH have been thoroughly investigated in human [16,17] and animal populations [18], and in this study were estimated after reconstruction of haplotypes via GFPS. Thus, only haplotypes made up by 20 or more identical SNP alleles for each two individuals were used. ROIs were used to estimate patterns of Mendelian segregation as well as genetic relatedness between two individuals due to the inheritance of paternal or maternal gametes.

### Monte-Carlo computer simulation of a phased chromosome

A Monte-Carlo computer simulation of BTA1 using phasing results of the cattle family was performed. A total number of progeny was 36. Only heterozygous SNPs (for the sire) were simulated (sire homozygous SNPs are trivial for phasing purposes). The chromosome coming from the sire to their calves was assumed to come from meiosis with a probability of 0, 1, 2, or 3 recombination events of 0.511494, 0.398467, 0.081418, and 0.00862, respectively. These are the genome-wide values found for recombination events per chromosome in our study. Once, the two resulting gametes from the sires were created then a drawing from the uniform distribution was used to assign either of the two chromosomes to a calf. The chromosome coming from the dams was generated using the haplotype frequencies (of chromosome 1) of the two first SNPs as estimated in this study. The next SNP was simulated using haplotype frequencies (for SNPs second and third) but conditional to the SNP allele at the second SNP. This process was repeated until the entire chromosome was terminated. The same procedure was followed to generate BTA1 for each of the 36 calves. The SNP data was randomly allocate to disturb the order of alleles and the resulting data was analyzed with the methods developed in this paper. The location of each (base pair) was the same as SNPs in the real data. Also, markers with MAF<0.10 were excluded. A total of 7,000 replicates were performed.

## Results

### Monte Carlo computer results from joint estimation of recombination fraction and linkage disequilibrium

**Figure 2:**
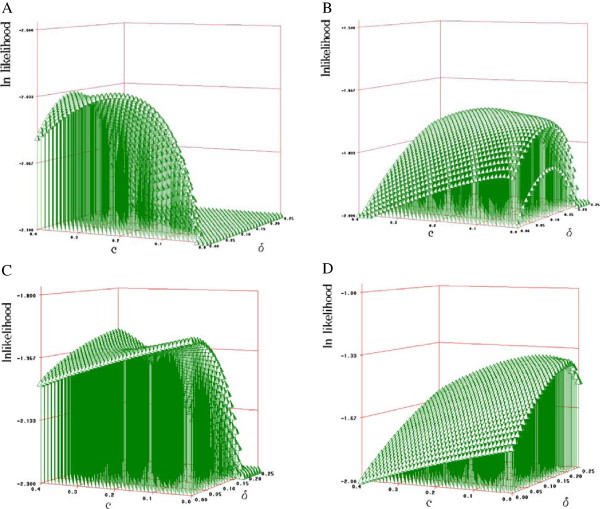
**Three-dimensional plot of ln of the likelihood (equation A1) along recombination fraction (c) and linkage disequilibrium (*δ*) when testing a family of infinite size for different situations regarding c and *δ*.** The allele frequencies were intermediate at the two loci. **A)** Situations simulated is c=0.20, *δ*=0. The maximum value was c=0.20, *δ*=0. **B) **Situations simulated is c=0.20, *δ*=0.20. The maximum value was at c=0.10, *δ*=0.15. **C) **Situations simulated is c=0.00, *δ*=0.00. The maximum value was c=0.00, *δ*=0.00. **D) **Situations simulated is c=0.00, *δ*=0.20. The maximum value was c=0.00, *δ*=0.20.

An E.M. algorithm was implemented for the joint estimation of recombination fraction and linkage disequilibrium in both single and multiple family situations (Appendix 1). Tables 1 and 2 show the results for the joint estimation of recombination fraction in a half-sib family with varying family sizes (36 to 2,000). The results show that both recombination fraction and linkage disequilibrium are accurately estimated when either true recombination or true disequilibrium is 0 even for relative small family sizes (36). On the contrary, estimates tend to be biased when both recombination fraction and linkage disequilibrium (absolute value) are greater than 0. Substituting the observed counts in equation A1 by their expected values according to true parameters (recombination fraction, linkage disequilibrium, allele frequencies) was carried out to plot the log of the likelihood against those parameters in order to investigate if the maximum likelihood method can or cannot separate effects of recombination fraction and linkage disequilibrium (i.e., behavior of the likelihood when sample size is infinite). Figure 2 shows several scenarios (A to D) regarding true parameters. If the method would work properly then the highest peak (maximum value of the likelihood) should correspond to true parameters. If either true recombination fraction or linkage disequilibrium is 0 then there is one single maximum value, which corresponds to the true parameters (Figure 2A, C and D). If the true values of both recombination fraction and linkage disequilibrium are different from 0 then estimates of both parameters are biased (Figure 2B). This occurs because the estimation of both parameters is confounded. Nevertheless, estimation of recombination fraction would be unbiased if true recombination is 0, which is necessary for phasing parents.

**Table 2:** **Average estimates of recombination fraction in a double heterozygote sire with varying half-sib family size and varying recombination fraction (*c*) and linkage disequilibrium (*δ*)**

		**Family size**					
**Simulated *c***	**Simulated *δ***	**36**	**100**	**300**	**500**	**1,000**	**2,000**
**0.00**	-0.10	0.0007 (0.1232)	0.0000 (0.0000)	0.0000 (0.0000)	0.0000 (0.0000)	0.0000 (0.0000)	0.0000 (0.0000)
	0.00	0.0000 (0.0031)	0.0000 (0.0000)	0.0000 (0.0000)	0.0000 (0.0000)	0.0000 (0.0000)	0.0000 (0.0000)
	0.05	0.0000 (0.0000)	0.0000 (0.0000)	0.0000 (0.0000)	0.0000 (0.0000)	0.0000 (0.0000)	0.0000 (0.0000)
	0.10	0.0000 (0.0000)	0.0000 (0.0000)	0.0000 (0.0000)	0.0000 (0.0000)	0.0000 (0.0000)	0.0000 (0.0000)
	0.20	0.0000 (0.0000)	0.0000 (0.0000)	0.0000 (0.0000)	0.0000 (0.0000)	0.0000 (0.0000)	0.0000 (0.0000)
**0.10**	-0.10	0.1046 (0.1080)	0.0996 (0.0518)	0.0998 (0.0284)	0.0997 (0.0156)	0.0997 (0.0156)	0.0999 (0.0111)
	0.00	0.1212 (0.1444)	0.1098 (0.0881)	0.1016 (0.0398)	0.1008 (0.2290)	0.1002 (0.0204)	0.1002 (0.0145)
	0.05	0.1157 (0.1425)	0.1184 (0.1033)	0.1078 (0.0603)	0.1034 (0.0412)	0.1011 (0.0259)	0.1007 (0.0176)
	0.10	0.0926 (0.1262)	0.1168 (0.1029)	0.1154 (0.0733)	0.1122 (0.0605)	0.1065 (0.0427)	0.1029 (0.0277)
	0.20	0.0227 (0.0550)	0.0459 (0.0639)	0.0740 (0.0541)	0.0732 (0.0462)	0.0770 (0.0369)	0.0808 (0.0299)
**0.20**	-0.10	0.2148 (0.1510)	0.2034 (0.0770)	0.2007 (0.0382)	0.2000 (0.0292)	0.2001 (0.0206)	0.2001 (0.0145)
	0.00	0.2167 (0.1670)	0.2212 (0.1204)	0.216 (0.0749)	0.2055 (0.0536)	0.2019 (0.0336)	0.2009 (0.0221)
	0.05	0.1970 (0.1607)	0.2110 (0.1172)	0.2152 (0.0841)	0.2131 (0.0703)	0.2094 (0.0541)	0.2052 (0.0373)
	0.10	0.1627 (0.1493)	0.1868 (0.1087)	0.1986 (0.0778)	0.2007 (0.0663)	0.2044 (0.0551)	0.2069 (0.0460)
	0.20	0.0568 (0.0959)	0.0944 (0.0905)	0.1040 (0.0663)	0.1061 (0.0573)	0.1068 (0.0467)	0.1075 (0.0388)

The simulated allele frequencies were ƒ_*T *_= 0.5 and ƒ_*M *_= 0.5. The number of replicates was 10^4^. The values between parentheses are standard deviations among replicates.

Table 3 shows the average of the estimate of the recombination fraction after assuming linkage equilibrium as described by [19] and as it has been assumed for all published QTL mapping experiments [20]. It is shown that estimates of the recombination fraction are generally biased even for true recombination fraction of 0 when family size is small. Table 4 shows computer simulation results for multi-family joint estimation of recombination fraction and linkage disequilibrium. The results show that estimates of both linkage disequilibrium and recombination fraction are unbiased in this setting regardless of the true (simulated) value of those parameters.

**Table 3:** **Average estimates of recombination fraction assuming linkage equilibrium**[19]**in a double heterozygote sire with varying half-sib family size and varying recombination fraction (*c*) and linkage disequilibrium (*δ*)**

		**Family size**					
**Simulated *c***	**Simulated *δ***	**36**	**100**	**300**	**500**	**1,000**	**2,000**
**0.00**	-0.10	0.0634 (0.1460)	0.0053 (0.0325)	0.0000 (0.0009)	0.0000 (0.0000)	0.0000 (0.0000)	0.0000 (0.0000)
	0.00	0.0123 (0.0566)	0.0002 (0.0057)	0.0000 (0.0000)	0.0000 (0.0000)	0.0000 (0.0000)	0.0000 (0.0000)
	0.05	0.0044 (0.0286)	0.0001 (0.0026)	0.0000 (0.0000)	0.0000 (0.0000)	0.0000 (0.0000)	0.0000 (0.0000)
	0.10	0.0012 (0.0124)	0.0000 (0.0003)	0.0000 (0.0000)	0.0000 (0.0000)	0.0000 (0.0000)	0.0000 (0.0000)
	0.20	0.0000 (0.0012)	0.0000 (0.0000)	0.0000 (0.0000)	0.0000 (0.0000)	0.0000 (0.0000)	0.0000 (0.0000)
**0.10**	-0.10	0.2620 (0.1938)	0.2259 (0.1181)	0.2108 (0.0580)	0.2085 (0.0444)	0.2067 (0.0313)	0.2066 (0.0220)
	0.00	0.1250 (0.1360)	0.1042 (0.0636)	0.1017 (0.0350)	0.1009 (0.0190)	0.1002 (0.0190)	0.1002 (0.0135)
	0.05	0.0814 (0.1006)	0.0710 (0.0492)	0.0698 (0.0274)	0.0694 (0.0212)	0.0691 (0.0149)	0.0692 (0.0106)
	0.10	0.0510 (0.0721)	0.0463 (0.0373)	0.0457 (0.0210)	0.0457 (0.0163)	0.0455 (0.0116)	0.0456 (0.0082)
	0.20	0.0123 (0.0293)	0.0119 (0.0171)	0.0126 (0.0097)	0.0124 (0.0077)	0.0124 (0.0053)	0.0124 (0.0040)
**0.20**	-0.10	0.3788 (0.1609)	0.3889 (0.1139)	0.3793 (0.0731)	0.3727 (0.0541)	0.3692 (0.0352)	0.3689 (0.0245)
	0.00	0.2343 (0.0764)	0.2068 (0.0876)	0.2024 (0.0470)	0.2010 (0.0359)	0.2003 (0.0254)	0.2004 (0.0181)
	0.05	0.1652 (0.1410)	0.1459 (0.0691)	0.1442 (0.0387)	0.1433 (0.0296)	0.1429 (0.0210)	0.1431 (0.0150)
	0.10	0.1078 (0.1086)	0.0981 (0.0546)	0.0973 (0.0308)	0.0970 (0.0238)	0.0967 (0.0168)	0.0968 (0.0112)
	0.20	0.0285 (0.0486)	0.0270 (0.0270)	0.0272 (0.0152)	0.0268 (0.0123)	0.0270 (0.0085)	0.0271 (0.0061)

The simulated allele frequencies were ƒ_*T *_= 0.5 and ƒ_*M *_= 0.5.The number of replicates was 10^4^. The values between parentheses are standard deviations among replicates.

**Table 4:** **Average of the estimates of linkage disequilibrium (*δ*) and recombination fraction (*c*) using the E.M. algorithm for joint estimation in multiple half-sib families**

	**Simulated *c***					
**Simulated *δ***	**0**		**0.25**		**0.50**	
	***δ***	***c***	***δ***	***c***	***δ***	***c***
**0.000**	0.0002 (0.0165)	0.0001 (0.0042)	0.0002 (0.0165)	0.2563 (0.0816)	0.0002 (0.0165)	0.5017 (0.0937)
**0.010**	0.0102 (0.0165)	0.0002 (0.0052)	0.0102 (0.0165)	0.2560 (0.0827)	0.0102 (0.0165)	0.5013 (0.0935)
**0.020**	0.0202 (0.0164)	0.0003 (0.0059)	0.0202 (0.164)	0.2557 (0.0835)	0.0202 (0.0164)	0.5002 (0.0930)
**0.030**	0.0301 (0.0164)	0.0007 (0.0093)	0.0301 (0.0165)	0.2554 (0.0841)	0.0301 (0.1646)	0.5000 (0.0922)
**0.040**	0.0400 (0.0163)	0.0011 (0.0112)	0.0400 (0.0163)	0.2550 (0.0840)	0.0400 (0.0163)	0.4993 (0.0905)
**0.050**	0.0499 (0.0163)	0.0016 (0.0133)	0.4999 (0.0163)	0.2547 (0.0827)	0.0499 (0.0163)	0.4991 (0.0887)
**0.075**	0.0748 (0.0160)	0.0043 (0.0201)	0.0748 (0.0160)	0.2539 (0.0787)	0.0748 (0.0160)	0.4990 (0.0812)
**0.100**	0.0998 (0.0155)	0.0079 (0.0256)	0.0997 (0.0154)	0.2529 (0.0730)	0.0997 (0.0155)	0.4989 (0.0732)
**0.125**	0.01245 (0.0146)	0.0107 (0.0267)	0.1245 (0.0146)	0.2507 (0.0658)	0.1245 (0.0146)	0.4985 (0.0646)
**0.150**	0.1494 (0.0136)	0.0135 (0.0269)	0.1494 (0.0136)	0.2500 (0.0585)	0.1494 (0.0136)	0.4991 (0.0577)
**0.175**	0.1743 (0.0122)	0.0145 (0.0249)	0.1743 (0.0122)	0.2500 (0.0508)	0.1743 (0.0122)	0.4994 (0.0507)
**0.200**	0.1991 (0.0102)	0.0133 (0.0203)	0.1991 (0.0102)	0.2497 (0.0430)	0.1991 (0.0102)	0.4996 (0.0445)
**0.250**	0.2489 (0.0020)	0.0000 (0.0000)	0.2488 (0.0020)	0.2498 (0.0289)	0.2489 (0.0020)	0.5001 (0.0339)

The simulated allele frequencies were ƒ_*T *_= 0.5 and ƒ_*M *_= 0.5. The simulation was carried out for varying recombination fractions, linkage disequilibria and resembling the Norwegian cattle population structure. The number of replicates was 10^4^. The values between parentheses are the average of standard deviations of the estimates of *δ *and *c*.

### Genome fragmentation phasing strategy in a cattle half-sib family

**Figure 3:**
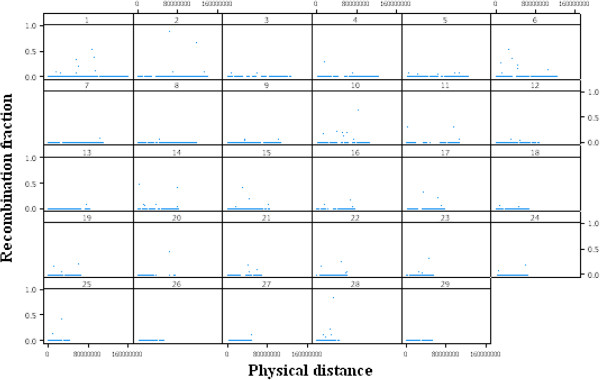
Genome-wide estimates of the recombination fraction between consecutive SNP during fragmentation along physical distance for the 29 autosomal chromosomes.

**Figure 4:**
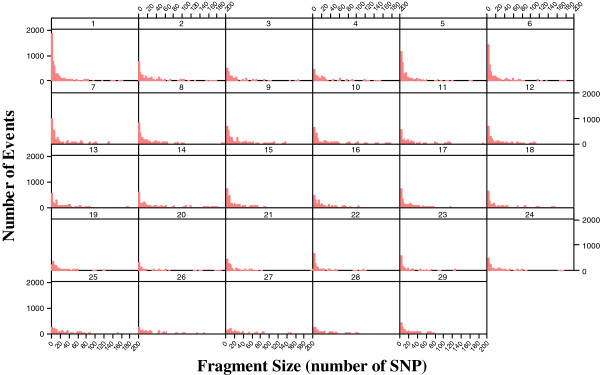
Histograms of the number of fragments for the 29 autosomal chromosomes according to fragment’s size (number of SNPs).

**Figure 5:**
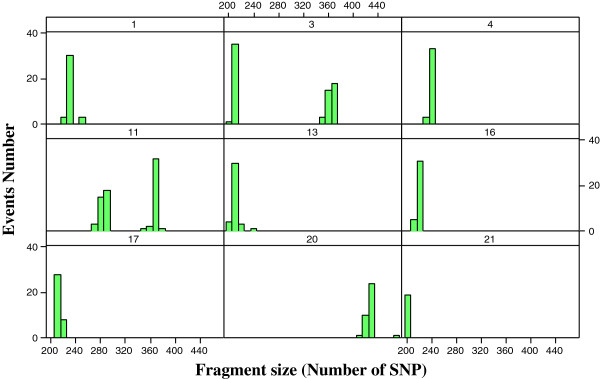
Histograms of the number of fragments for the autosomal chromosomes that have 200 or more SNPs (cold spots) per fragment for autosomes 1,3,4,11,13,16,17,20, and 21.

A method named genome fragmentation phasing strategy was developed for phasing parents and progeny and used for phasing a cattle family comprising 36 calves to illustrate GFPS. The first step was to estimate recombination fraction between each of two consecutive SNPs. The genome-wide distribution of recombination events between each of two consecutive SNPs is depicted in Figure 3. Although the great majority (over 92%) of the estimates were 0.00, there were many estimates of recombination fraction too high for the physical distance separating them. It may be attributed to either miss location of SNPs during the sequencing and alignment or genotyping errors. A recombination fraction larger than 0 was used (as a signal) to terminate a fragment and to initiate the next one. The fragmentation yielded a distribution of fragment size across the genome (number of SNPs per fragment) shown in Figure 4. Most of the fragments were rather small but some relatively large fragments (more than 200 SNPs) allowed genome-wide identification of cold spots (Figure 5). These cold spots are for the sire producing meiosis and if tested in multiple families would allow distinguishing between cold spots at the individual and population levels.

**Figure 6:**
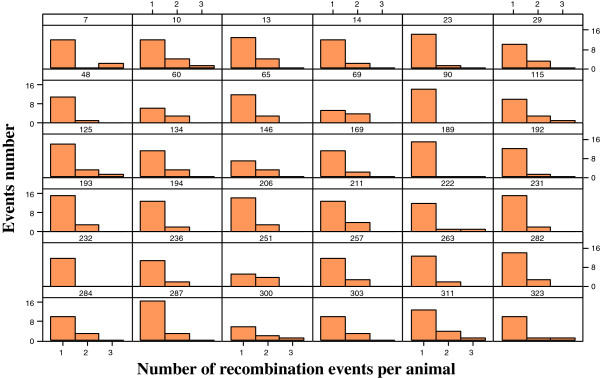
Distribution of the number of recombination events for the 36 calves in a half-sib family.

**Figure 7:**
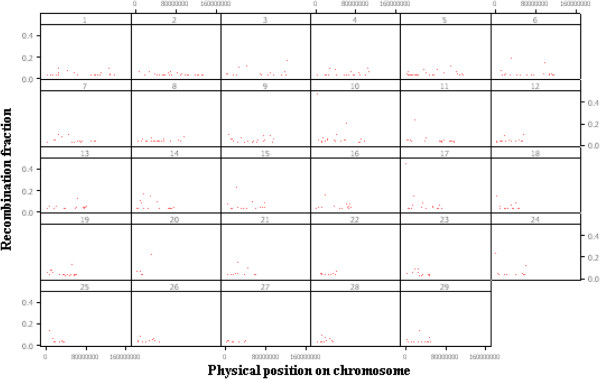
Estimates of recombination fraction between fragments across the 29 autosomal chromosomes.

The number of recombination events per chromosome per individual (gamete) is shown in Figure 6. There were some calves (189 and 284) with only single recombination event per chromosome. The distribution of all recombination events between fragments generated by GFPS along the 29 autosomes is given in Figure 7. There were 613 recombination events detected. In some situations, recombinations are evenly distributed in the genome. However, some areas have higher values suggesting either hot spots or miss location of whole fragments during the process of sequencing and assembling to produce Illumina’s array. For example, recombination fraction between the first fragment in chromosome 17 (unlinked with nearby fragments) was genome-wide tested (all fragments in the entire genome) and resulted in a recombination fraction estimate of 0.07 with another fragment in chromosome 19 which suggests an error of assembling in the Illumina array.

The informativity of SNPs greatly increased after haplotype reconstruction using GFPS. More than 90% (range 86.1 to 95.1% for the 29 chromosomes) of non-informative SNPs become informative due to the use of the information of SNPs linked to them.

**Figure 8:**
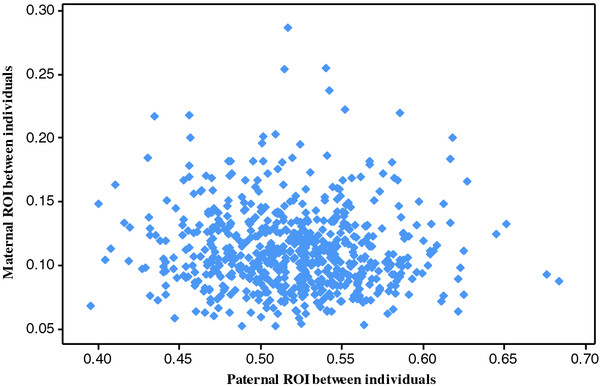
**Paternal versus maternal ROIs for all combinations of a pair of half-sibs.** There were 36 calves which makes 630 combinations (points in the graph).

**Figure 9:**
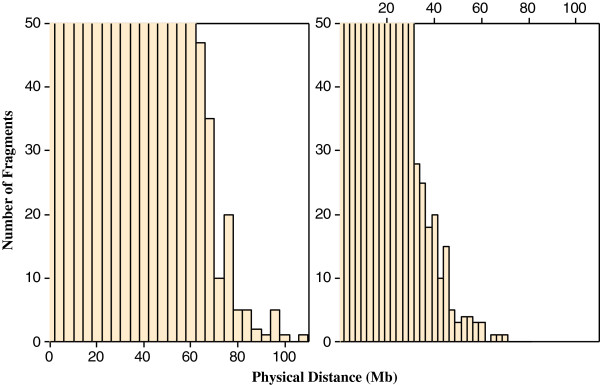
**Histograms of the distribution of fragments that are ROIs for any pair of individuals and with size larger than 10 Mb. **The histogram represents the distribution of ROIs of origin paternal (left) and maternal (right).

The analysis of genome regions of identity shared by two individuals is given in Figure 8. The analyses compare regions of identity (ROIs) inherited from sire or from dam for each of two individuals in a half-sib family. As expected for half-sibs, the regions shared or identical from paternal origin between two individuals is on average 0.52, with a range of values between 0.40 and 0.68. The maternally inherited ROIs between individuals ranged between 0.05 and 0.29 with a mean of 0.11. These results suggest that there is a significant amount of fragments with a relative large variation that are identical by descent among unrelated dams. Figure 9 shows the tail of the distributions of paternal and maternal ROIs. Fragments of more than 20 Mb of maternal autosomes are commonly shared by individuals with unrelated mothers.

The results of the simulation of BTA1 with the same LD and genetic linkage structure of our data are depicted in Table 5. The results of correct phasing are for informative segments. That is, homozygote calves allowed identification of origin of haplotype (paternal or maternal). Non informative areas are detectable and cannot be phased out. The phasing method was very accurate to identify haplotypes in both sires and calves. Recombination events were considered identified if they were less that 3Mb apart from the true (simulated) event. The average distance between the true (simulated) and identified recombination events was 0.79 Mb.

**Table 5:** Monte-Carlo simulation results of BTA1 with the same LD and genetic linkage observed in the real data

**Average over replicates**	
Total number of SNPs in BTA1	3,343
Total number of SNPs bull heterozygote	751
Percentage of correct phases in informative segments in sire	99.98%
Percentage of correct identification of recombination events	99.87%
Average distance between true and estimated position for recombination events	0.793 Mb (0.002)
Percentage of correct phases in informative segments in calves	99.94%

Informative SNPs are those that can be traced up to sire or dam.

## Discussion

This paper describes a new method, GFPS, for reconstructing haplotypes after the use of SNP arrays in breeding populations in which large groups of progeny from at least one progenitor are available. The method permits reconstruction of long haplotypes utilizing consecutive SNPs within a fragment in a similar fashion to the process of sequencing and assembling small fragments of DNA that are aligned using common sequences in the extremes in order to generate larger fragments. In turn, linkage analyses of long fragments can identify recombination events and pinpoint genotyping or assembling errors. The application of this method provides a new insight into the genome of highly used sires or dams by allowing identification of individual hot and cold spots or identifying chromosomes with non Mendelian segregation which might be caused by chromosomal abnormalities. The application of this method allows a high control of the haplotypes passing from generation to generation and can provide a better understanding of the genetic basis of production and diseases in breeding populations.

The alternative to the proposed GFPS is long-range phasing and imputation methods [21,22] which make use of surrogate fathers and mothers in order to identify haplotypes. In general, imputation methods utilize allele frequencies in order to reconstruct haplotypes by assuming that the larger the allele frequencies, the larger the chance of being represented in the haplotypes. Consequently, there is always an error associated to imputed haplotypes and rare alleles are missed. The advantage of the method presented here and described elsewhere is that information on sibs is used together with the laws of inheritance [5,10]. The novelty of our method is that it includes joint estimation of recombination and LD for reconstruction of parents and progeny haplotypes. As shown in this paper, if only one parent is available then both parameters can be accurately estimated if at least one of them is 0 as has been shown here with Monte Carlo computer simulations. Homozygous progeny from heterozygous sires flag which allele and haplotype is inhered from the sire, and consequently, allow identification of haplotypes of paternal and maternal origin. It dramatically increases informativity of heterozygous SNPs between parent and progeny (over 90%).

Many of the applications of SNP arrays in farm animals ignore the sequential nature of polymorphism in haplotypes. For example, in many instances genomic selection [1] assumes a large number of unlinked loci. As discussed in previous work it has implications in genomic evaluations that are systematically ignored [2,3,23]: a) progeny from the same parent share large haplotypes (not just single SNP) as shown with ROIs in this paper, and b) single heterozygous SNPs for both sire and half-sib progeny are non-informative, and consequently information is reduced or lost in genomic evaluations. Other advantages and potential applications of the proposed method are: 1) requires only currently available genotypic information on progeny as required for genomic selection when using juveniles for shortening generation intervals, 2) can be applied to any farm animal with large families even if only one parent is common to a group of progeny, 3) facilitates haplotyping the entire breeding population for new investigations such as signatures of selection or high order linkage disequilibrium, 4) can be used to estimate molecular relatedness more precisely by using haplotypes of given length rather than the sum of single SNPs (large fragments are likely identical by descent), and 5) can help to tracing up allele and haplotypes through generations which may facilitate the detection of genes involved in diseases or production.

The methods developed are designed for haplotyping farm animals with large families. However, they can also be applied to wild animals as long as large families and dense SNP arrays are available in those species. After all, the cattle used in our study came from a free range production system where the only samples or records taken were from DNA which was first used for paternity testing and then for phasing haplotypes.

## Conclusions

Haplotype phasing is possible and highly accurate when estimating jointly linkage disequilibrium and genetic linkage in animal breeding populations as long as large families are available.

## Appendix 1

### Joint estimation of genetic linkage and linkage disequilibrium in half-sib families

Let the sire have genotype *TtMm* at two SNPs, *T/t*, and *M/m* and linkage phase (*TM/tm*) and *n*_*j,i*_ be the genotype counts from offspring from the *i-th* sire family (*j*=*TTMM*, *TTMm*, *TTmm*, *TtMM*, *TtMm*, *Ttmm, ttMM, ttMm,* and *ttmm*). The recombination fraction, *c,* is estimated simultaneously with linkage disequilibrium (*δ*), and allele frequencies (*f*_*T*_*, f*_*M*_). The likelihood equation for linkage phase TM/tm for data of the *i-th* half-sib family is:

(A1) $$\begin{array}{c} L_{\mathrm{TM}/\mathrm{tm},i}\left(\delta ,f_{T},f_{M},c|\;\mathrm{nG}\right)=K{\left(\phi_{\text{TTMm}}\right)}^{n_{\text{TTMm},i}}{\left(\phi_{\text{TTMm}}\right)}^{n_{\text{TTMm},i}}\\ \;{\left(\phi_{\text{TTMm}}\right)}^{n_{\text{TTMm},i}}{\left(\phi_{\text{TTMm}}\right)}^{n_{\text{TTMm},i}}{\left(\phi_{\text{TTMm}}\right)}^{n_{\text{TTMm},i}}\\ \;{\left(\phi_{\text{TTMm}}\right)}^{n_{\text{TTMm},i}}{\left(\phi_{\text{TTMm}}\right)}^{n_{\text{TTMm},i}}{\left(\phi_{\text{TTMm}}\right)}^{n_{\text{TTMm},i}}{\left(\phi_{\text{TTMm}}\right)}^{n_{\text{TTMm},i}} \end{array}$$

where the probabilities of offspring genotypes among half-sib offspring are obtained from Table 6: ϕTTMm=121-cfTM; ϕTTMm=121-cfTm+12cfTM; ϕTTMm=12cfTm; ϕTTMm=121-cftM+12cfTM; ϕTTMm=121-cftm+fTM+12cftM+fTm; ϕTTMm=121-cfTm+12cftm; ϕTTMm=12cftMϕTTMm=121-cftM+12cftm; and ϕTTMm=121-cftm.

**Table 6:** Genotypes and their frequencies among half-sib progeny from a double heterozygote sire

	**Sire (phase TM/tm)**			
**Dam**	**TM**	**Tm**	**tM**	**tm**
G freq	½ (1-c)	½ c	½ c	½ (1-c)
TM f_TM_	TTMM ½ (1-c) f_TM_	TTMm ½ c f_TM_	TtMM ½ c f_TM_	TtMm ½ (1-c) f_TM_
Tm f_Tm_	TTMm ½ (1-c) f_Tm_	TTmm ½ c f_Tm_	TtMm ½ c f_Tm_	Ttmm ½ (1-c) f_Tm_
tM f_tM_	TtMM ½ (1-c) f_tM_	TtMm ½ c f_tM_	ttMM ½ c f_tM_	ttMm ½ (1-c) f_tM_
tm f_tm_	TtMm ½ (1-c) f_tm_	Ttmm ½ c f_tm_	ttMm ½ c f_tm_	ttmm ½ (1-c) f_tm_
	**Sire (phase Tm/tM)**			
**Dam**	**TM**	**Tm**	**tM**	**tm**
G freq	½ c	½ (1-c)	½ (1-c)	½ c
TM f_TM_	TTMM ½ c f_TM_	TTMm ½ (1-c)f_TM_	TtMM ½ (1-c)f_TM_	TtMm ½ c f_TM_
Tm f_Tm_	TTMm ½ c f_Tm_	TTmm ½ (1-c)f_Tm_	TtMm ½ (1-c)f_Tm_	Ttmm ½ c f_Tm_
tM f_tM_	TtMM ½ c f_tM_	TtMm ½ (1-c)f_tM_	ttMM ½ (1-c)f_tM_	ttMm ½ c f_tM_
tm f_tm_	TtMm ½ c f_tm_	Ttmm ½ (1-c)f_tm_	ttMm ½ (1-c)f_tm_	ttmm ½ c f_tm_

*G *gametes, *freq *frequency.

Likelihood equation [A1] can be solved by applying the E.M algorithm:

(A2) $$\begin{array}{ll} {\hat{f}}_{\mathrm{TM}}^{i}=\frac{1}{N_{i}}(n_{\text{TTMM,i}}+\frac{c{\hat{f}}_{\mathrm{TM}}^{i}n_{\text{TTMm},i}}{c{\hat{f}}_{\mathrm{TM}}^{i}+\left(1-c\right){\hat{f}}_{\mathrm{Tm}}^{i}}+\frac{c{\hat{f}}_{\mathrm{TM}}^{i}n_{\text{TtMM},i}}{c{\hat{f}}_{\mathrm{TM}}^{i}+\left(1-c\right){\hat{f}}_{\mathrm{tM}}^{i}}\\ \;+\frac{\left(1-c\right){\hat{f}}_{\mathrm{TM}}^{i}n_{\text{TtMm},i}}{\left[c\left({\hat{f}}_{\mathrm{Tm}}^{i}+{\hat{f}}_{\mathrm{tM}}^{i}\right)+\left(1-c\right)\left({\hat{f}}_{\mathrm{TM}}^{i}+{\hat{f}}_{\mathrm{tm}}^{i}\right)\right]})\\ {\hat{f}}_{\mathrm{Tm}}^{i}=\frac{1}{N_{i}}(n_{\text{TTmm,i}}+\frac{\left(1-c\right){\hat{f}}_{\mathrm{Tm}}^{i}n_{\text{TTMm},i}}{c{\hat{f}}_{\mathrm{TM}}^{i}+\left(1-c\right){\hat{f}}_{\mathrm{Tm}}^{i}}+\frac{\left(1-c\right){\hat{f}}_{\mathrm{Tm}}^{i}n_{\text{Ttmm},i}}{c{\hat{f}}_{\mathrm{tm}}^{i}+\left(1-c\right){\hat{f}}_{\mathrm{Tm}}^{i}}\\ \;+\frac{c{\hat{f}}_{\mathrm{Tm}}^{i}n_{\text{TtMm},i}}{\left[c\left({\hat{f}}_{\mathrm{Tm}}^{i}+{\hat{f}}_{\mathrm{tM}}^{i}\right)+\left(1-c\right)\left({\hat{f}}_{\mathrm{TM}}^{i}+{\hat{f}}_{\mathrm{tm}}^{i}\right)\right]})\\ {\hat{f}}_{\mathrm{tM}}^{i}=\frac{1}{N_{i}}(n_{\text{ttMM,i}}+\frac{\left(1-c\right){\hat{f}}_{\mathrm{tM}}^{i}n_{\text{TtMM},i}}{c{\hat{f}}_{\mathrm{TM}}^{i}+\left(1-c\right){\hat{f}}_{\mathrm{tM}}^{i}}+\frac{\left(1-c\right){\hat{f}}_{\mathrm{tM}}^{i}n_{\text{ttMm},i}}{c{\hat{f}}_{\mathrm{tm}}^{i}+\left(1-c\right){\hat{f}}_{\mathrm{tM}}^{i}}\\ \;+\frac{c{\hat{f}}_{\mathrm{tM}}^{i}n_{\text{TtMm},i}}{\left[c\left({\hat{f}}_{\mathrm{Tm}}^{i}+{\hat{f}}_{\mathrm{tM}}^{i}\right)+\left(1-c\right)\left({\hat{f}}_{\mathrm{TM}}^{i}+c{\hat{f}}_{\mathrm{tm}}^{i}\right)\right]})\\ {\hat{f}}_{\mathrm{tm}}^{i}=\frac{1}{N_{i}}(n_{\text{ttmm,i}}+\frac{c{\hat{f}}_{\mathrm{tm}}^{i}n_{\text{Ttmm},i}}{c{\hat{f}}_{\mathrm{tm}}^{i}+\left(1-c\right){\hat{f}}_{\mathrm{Tm}}^{i}}+\frac{c{\hat{f}}_{\mathrm{tm}}^{i}n_{\text{ttMm},i}}{c{\hat{f}}_{\mathrm{tm}}^{i}+\left(1-c\right){\hat{f}}_{\mathrm{tM}}}\\ \;+\frac{\left(1-c\right){\hat{f}}_{\mathrm{tm}}^{i}n_{\text{ttMm},i}}{\left[c\left({\hat{f}}_{\mathrm{Tm}}^{i}+{\hat{f}}_{\mathrm{tM}}^{i}\right)+\left(1-c\right)\left({\hat{f}}_{\mathrm{TM}}^{i}+{\hat{f}}_{\mathrm{tm}}^{i}\right)\right]})\\ {\hat{c}}^{i}=\frac{1}{N_{i}}(n_{\text{TTmm,i}}+n_{\text{ttMM,i}}+\frac{c{\hat{f}}_{\mathrm{TM}}^{i}n_{\text{TTMm},i}}{\left(1-c\right){\hat{f}}_{\mathrm{TM}}^{i}+c{\hat{f}}_{\mathrm{Tm}}^{i}} & \\ +\frac{c{\hat{f}}_{\mathrm{TM}}^{i}n_{\text{TtMM},i}}{c{\hat{f}}_{\mathrm{TM}}^{i}+\left(1-c\right){\hat{f}}_{\mathrm{tM}}^{i}}+\frac{c\left({\hat{f}}_{\mathrm{Tm}}^{i}+{\hat{f}}_{\mathrm{tM}}^{i}\right)n_{\text{TtMm},i}}{\left[c\left({\hat{f}}_{\mathrm{Tm}}^{i}+{\hat{f}}_{\mathrm{tM}}^{i}\right)+\left(1-c\right)\left({\hat{f}}_{\mathrm{TM}}^{i}+{\hat{f}}_{\mathrm{tm}}^{i}\right)\right]}\\ \;+\frac{c{\hat{f}}_{\mathrm{tm}}^{i}n_{\text{Ttmm},i}}{c{\hat{f}}_{\mathrm{tm}}^{i}+\left(1-c\right){\hat{f}}_{\mathrm{Tm}}^{i}}+\frac{c{\hat{f}}_{\mathrm{tm}}^{i}n_{\text{ttMm},i}}{c{\hat{f}}_{\mathrm{tm}}^{i}+\left(1-c\right){\hat{f}}_{\mathrm{tM}}^{i}}) \end{array}$$

where *N*_*i*_ is the size of the *i-th* half-sib family. Using initial values of the haplotype frequencies and iterating over equation A2 will converge to ML estimates of haplotype frequencies. Linkage disequilibrium is estimated by $\hat{\delta }={\hat{f}}_{\mathrm{TM}}^{i}{\hat{f}}_{\mathrm{tm}}^{i}-{\hat{f}}_{\mathrm{Tm}}^{i}{\hat{f}}_{\mathrm{tM}}^{i}$.

If the linkage phase of the sire is Tm/tM then the E.M. equations are:

$$\begin{array}{l} {\hat{f}}_{\mathrm{TM}}^{i}=\frac{1}{N_{i}}(n_{\text{TTMM},i}+\frac{\left(1-c\right){\hat{f}}_{\mathrm{TM}}^{i}n_{\text{TTMm,i}}}{\left(1-c\right){\hat{f}}_{\mathrm{TM}}^{i}+c{\hat{f}}_{\mathrm{Tm}}^{i}}\\ \;+\frac{\left(1-c\right){\hat{f}}_{\mathrm{TM}}^{i}n_{\text{TtMM,i}}}{\left(1-c\right){\hat{f}}_{\mathrm{TM}}^{i}+c{\hat{f}}_{\mathrm{tM}}^{i}}\\ \;+\frac{c{\hat{f}}_{\mathrm{TM}}^{i}n_{\text{TtMm,i}}}{\left[\left(1-c\right)\left({\hat{f}}_{\mathrm{Tm}}^{i}+{\hat{f}}_{\mathrm{tM}}^{i}\right)+c\left({\hat{f}}_{\mathrm{TM}}^{i}+{\hat{f}}_{\mathrm{tm}}^{i}\right)\right]}) \end{array}$$

$$\begin{array}{l} {\hat{f}}_{\mathrm{Tm}}^{i}=\frac{1}{N_{i}}(n_{\text{TTmm},i}+\frac{c{\hat{f}}_{\mathrm{Tm}}^{i}n_{\text{TTMm,i}}}{\left(1-c\right){\hat{f}}_{\mathrm{TM}}^{i}+c{\hat{f}}_{\mathrm{Tm}}^{i}}\\ \;+\frac{c{\hat{f}}_{\mathrm{Tm}}^{i}n_{\text{Ttmm,i}}}{\left(1-c\right){\hat{f}}_{\mathrm{tm}}^{i}+c{\hat{f}}_{\mathrm{Tm}}^{i}}\\ \;+\frac{\left(1-c\right){\hat{f}}_{\mathrm{Tm}}^{i}n_{\text{TtMm,i}}}{\left[\left(1-c\right)\left({\hat{f}}_{\mathrm{Tm}}^{i}+{\hat{f}}_{\mathrm{tM}}^{i}\right)+c\left({\hat{f}}_{\mathrm{TM}}^{i}+{\hat{f}}_{\mathrm{tm}}^{i}\right)\right]}) \end{array}$$

$$\begin{array}{l} {\hat{f}}_{\mathrm{tM}}^{i}=\frac{1}{N_{i}}(n_{\text{ttMM},i}+\frac{c{\hat{f}}_{\mathrm{tM}}^{i}n_{\text{TtMM,i}}}{\left(1-c\right){\hat{f}}_{\mathrm{TM}}^{i}+c{\hat{f}}_{\mathrm{tM}}^{i}}\\ \;+\frac{c{\hat{f}}_{\mathrm{tM}}^{i}n_{\text{ttMm,i}}}{\left(1-c\right){\hat{f}}_{\mathrm{tm}}^{i}+c{\hat{f}}_{\mathrm{tM}}^{i}}\\ \;+\frac{\left(1-c\right){\hat{f}}_{\mathrm{tM}}^{i}n_{\text{TtMm,i}}}{\left[\left(1-c\right)\left({\hat{f}}_{\mathrm{Tm}}^{i}+{\hat{f}}_{\mathrm{tM}}^{i}\right)+c\left({\hat{f}}_{\mathrm{TM}}^{i}+{\hat{f}}_{\mathrm{tm}}^{i}\right)\right]}) \end{array}$$

$$\begin{array}{l} {\hat{f}}_{\mathrm{tm}}^{i}=\frac{1}{N_{i}}(n_{\text{ttmm},i}+\frac{\left(1-c\right){\hat{f}}_{\mathrm{tm}}^{i}n_{\text{Ttmm,i}}}{\left(1-c\right){\hat{f}}_{\mathrm{tm}}^{i}+c{\hat{f}}_{\mathrm{Tm}}^{i}}\\ \;+\frac{\left(1-c\right){\hat{f}}_{\mathrm{tm}}^{i}n_{\text{ttMm,i}}}{\left(1-c\right){\hat{f}}_{\mathrm{tm}}^{i}+c{\hat{f}}_{\mathrm{tM}}^{i}}\\ \;+\frac{c{\hat{f}}_{\mathrm{tm}}^{i}n_{\text{TtMm,i}}}{\left[\left(1-c\right)\left({\hat{f}}_{\mathrm{Tm}}^{i}+{\hat{f}}_{\mathrm{tM}}^{i}\right)+c\left({\hat{f}}_{\mathrm{TM}}^{i}+{\hat{f}}_{\mathrm{tm}}^{i}\right)\right]}) \end{array}$$

$$\begin{array}{l} {\hat{c}}^{i}=\frac{1}{N_{i}}(n_{\text{TTMm},i}+n_{\text{ttmm},i}+\frac{c{\hat{f}}_{\mathrm{Tm}}^{i}n_{\text{TTMm,i}}}{\left(1-c\right){\hat{f}}_{\mathrm{TM}}^{i}+c{\hat{f}}_{\mathrm{Tm}}^{i}}\\ \;+\frac{c{\hat{f}}_{\mathrm{tM}}^{i}n_{\text{TtMM,i}}}{c{\hat{f}}_{\mathrm{tM}}^{i}+\left(1-c\right){\hat{f}}_{\mathrm{TM}}^{i}}\\ \;+\frac{c\left({\hat{f}}_{\mathrm{tm}}^{i}+{\hat{f}}_{\mathrm{TM}}^{i}\right)n_{\text{TtMm,i}}}{\left[c\left({\hat{f}}_{\mathrm{tm}}^{i}+{\hat{f}}_{\mathrm{TM}}^{i}\right)+\left(1-c\right)\left({\hat{f}}_{\mathrm{tM}}^{i}+{\hat{f}}_{\mathrm{Tm}}^{i}\right)\right]}\\ \;+\frac{c{\hat{f}}_{\mathrm{Tm}}^{i}n_{\text{Ttmm,i}}}{c{\hat{f}}_{\mathrm{Tm}}^{i}+\left(1-c\right){\hat{f}}_{\mathrm{tm}}^{i}}+\frac{c{\hat{f}}_{\mathrm{tM}}^{i}n_{\text{ttMm,i}}}{c{\hat{f}}_{\mathrm{tM}}^{i}+\left(1-c\right){\hat{f}}_{\mathrm{tm}}^{i}}) \end{array}$$

The probability of phase TM/tm is:

(A3) $$\begin{array}{l} \mathrm{Pr}\;\mathrm{of}\;\mathrm{Phase}\;\left(\mathrm{TM}/\mathrm{tm}\right)\;\\ \;=\frac{L_{\mathrm{TM}/\mathrm{tm}}\left(\delta ,f_{T},f_{M},c|\mathrm{data}\right)}{L_{\mathrm{TM}/\mathrm{tm}}\left(\delta ,f_{T},f_{M},c|\mathrm{data}\right)+L_{\mathrm{Tm}/\mathrm{tM}}\left(\delta ,f_{T},f_{M},c|\mathrm{data}\right)} \end{array}$$

where *L*_*TM*/*tm*_(*δ*, *f*_*T*_, *f*_*M*_, *c*|data) and *L*_*Tm*/*tM*_(*δ*, *f*_*T*_, *f*_*M*_, *c*|data) are the likelihoods of phase (*TM/tm*) and phase (*Tm/tM*) under a model estimating linkage disequilibrium (*δ*), allele frequencies for one of the alleles at markers *T/t* and *M/m* (*f*_*T*_, and *f*_*M*_), and the recombination fraction (*c*).

### Estimation of LD across multiple half-sib families

The likelihood equation to estimate LD across half-sib families is:

$$L\left(\delta ,f_{T},f_{M},c|\;\mathrm{nG}\right)=\prod_{i=1}^{\mathrm{nf}}L_{i}\left(\delta ,f_{T},f_{M},c|\;\mathrm{nG}\right)$$

where *L*_*i*_*(δ, f*_*T*_*, f*_*M*_*, c|nG)* is the likelihood for the *i-th* half-sib family conditional to genotype marker information (*nG*) and *nf* is the number of families. Note that depending on the sire genotype, allele frequencies for *T* and *M* (double homozygote) or *M* (homo-heterozygote) do not need to be estimated. The E.M. algorithm can be applied to multiple families by iterating on the four haplotype frequencies and recombination fraction:

(A4) $$\begin{array}{l} {\hat{f}}_{\mathrm{TM}}=\frac{\sum_{i=1}^{\mathrm{nf}}\left(N_{i}{\hat{f}}_{\mathrm{TM}}^{i}\right)}{\sum_{i=1}^{\mathrm{nf}}N_{i}},\\ {\hat{f}}_{\mathrm{Tm}}=\frac{\sum_{i=1}^{\mathrm{nf}}\left(N_{i}{\hat{f}}_{\mathrm{Tm}}^{i}\right)}{\sum_{i=1}^{\mathrm{nf}}N_{i}},\\ {\hat{f}}_{\mathrm{tM}}=\frac{\sum_{i=1}^{\mathrm{nf}}\left(N_{i}{\hat{f}}_{\mathrm{tM}}^{i}\right)}{\sum_{i=1}^{\mathrm{nf}}N_{i}},\\ {\hat{f}}_{\mathrm{tm}}=\frac{\sum_{i=1}^{\mathrm{nf}}\left(N_{i}{\hat{f}}_{\mathrm{tm}}^{i}\right)}{\sum_{i=1}^{\mathrm{nf}}N_{i}}\\ \hat{c}=\frac{\sum_{i=1}^{\mathrm{nf}}\left(N_{i}{\hat{c}}^{i}\right)}{\sum_{i=1}^{\mathrm{nf}}N_{i}} \end{array}$$

where equations for haplotype frequencies for each single family varies depending on the sire genotype (see [15] for a full description of all possible situations). Equation [A4] was solved iteratively after giving a starting value to the haplotype frequencies and by estimating in each iteration ${\hat{f}}_{T}={\hat{f}}_{\mathrm{Tm}}^{i}+{\hat{f}}_{\mathrm{TM}}^{i}$ and ${\hat{f}}_{M}={\hat{f}}_{\mathrm{TM}}^{i}+{\hat{f}}_{\mathrm{tM}}^{i}$.

## Abbreviations

SNP: Single Nucleotide Polymorphism; LD: Linkage disequilibrium; ROI: regions of identity; GFPS: Genome fragmentation phasing strategy.

## Competing interests

The authors declare that they have no competing interests.

## Authors’ contributions

LGR contributed to the developed the idea and carried out the mathematical modeling for the joint estimation of LD and linkage disequilibrium. He drafted the first version of the manuscript. AMH participated in the design of the study, contributed with preparation of DNA samples and writing of several parts of the manuscript. DT carried out the tissue sampling, and contributed to the writing of the manuscript. WMR contributed to the development of the idea, helped to carry out tissue sampling and contributed to the writing of the manuscript. All authors read and approved the final manuscript.
